# Feeling the clunk: Managing and attributing uncertainty in screening for developmental dysplasia of the hip in infancy

**DOI:** 10.1016/j.ssmqr.2022.100040

**Published:** 2022-12

**Authors:** Sarah Milton, Gill Gilworth, Andreas Roposch, Judith Green

**Affiliations:** a School of Life Course & Population Sciences, King's College London, London, UK; b Great Ormond Street Institute of Child Health, University College London, London, UK; c Wellcome Centre for Cultures & Environments of Health, University of Exeter, Exeter, UK

**Keywords:** Uncertainty, Developmental dysplasia of hip, Double contingency, Screening, Primary care, Infants

## Abstract

The management of uncertainty in clinical practice has been an enduring topic of sociological scholarship. However, little of this addresses how uncertainty and non-knowledge are attributed to the self and other actors. We take the example of checking for developmental dysplasia of the hip (DDH), part of infant screening in UK primary care, to examine the ‘double contingency’ of attributions of uncertainty and ignorance. Our data come from interviews with parents and General Practitioners (GPs), and observations of the six-week check conducted as part of a study to develop a checklist to aid GPs' diagnostic and referral decisions. Parents' pervasive uncertainties about managing with a new-born infant place them in a trusting relation to biomedicine, in which knowledge about infant hips is delegated to the clinical team: most described themselves as not-knowing about DDH. GPs focus on the uncertainties of applying sensory and experiential knowledge of infant bodies, in a consultation with more diffuse aims than screening for DDH. A prototype checklist, developed by orthopaedic specialists, was an explicit attempt to reduce uncertainty around thresholds for referral. However, using the checklist surfaced multiple logics of uncertainty. It also surfaced attributions of uncertainty and non-knowledge to other actors: orthopaedic specialists' assumptions about GPs' uncertain technical knowledge; GPs' assumptions about orthopaedic specialists' ignorance of the primary care setting; and clinicians' assumptions about the role of parental ignorance. This ‘double contingency’ of attributions of other actors' non-knowledge is a salient additional dimension to the uncertainty that infuses biomedical practice.

## Highlights

• We studied clinical uncertainty in screening for developmental dysplasia of the hip.
• A checklist for diagnosis in primary care revealed different logics of uncertainty.
• GPs focused on the inherent uncertainties of sensory and experiential knowledge.
• GPs, specialists and parents were oriented to the uncertainties of other actors.
• Doing medicine entails managing the ‘double contingency’ of uncertainty attribution.

## 1 Introduction

### 1.1 Uncertainty and evidence based medicine

Doing medicine entails managing multiple sources of uncertainty and their interactions: the inherent indeterminacies of biomedical knowledge; existential uncertainty about the future; and uncertainties about one's own clinical competence (Fox, 1980, 1989; Han 2011, Lian 2021). It is now well rehearsed that the application of ‘evidence-based medicine’ does not reduce these uncertainties in clinical practice, but rather reframes them, enabling new subjects of uncertainty and reconfiguring existing ones (Timmermans & Angell, 2001; Timmermans & Kolker, 2004; Armstrong, 2007). One arena in which these reconfigurations have been particularly potent is primary care. Primary care has a tradition of advocating for ‘patient-centred medicine’, which can sit uneasily with paradigms of evidence-based medicine that foreground routinized care through clinical guidelines and such (Bensing, 2000; Armstrong, 2007). The inherently unpredictable workload of primary care, with patients presenting with undifferentiated symptoms (Alam 2017; Armstrong, 2011), problematizes any straightforward application of evidence-based guidelines derived from population-level evidence applied to categories of patient. Drawing on chronic care management, May et al. (2006) suggest that clinical guidelines enter the primary care arena as ‘technogovernance’, a set of practices that enable (in principle) the heterogeneous elements of biomedicine to be contained, and managed, within the clinic. Guidelines and decision aids, they argue, can act as intermediaries between the different kinds of medical knowledge encapsulated in patient-centred and evidence-based medicine, with their “contradictory tendencies of subjective engagement and aggregated abstraction” (May et al., 2006: p1023). These technologies have the potential to distribute accountability and enact surveillance, through mechanisms such as decoupling individual experience from the clinical encounter, or providing a shared object (such as a guideline) that mediates the encounter between the patient's experience and abstract clinical knowledge. Inevitably, their operation is likely to be both partial and contested: the heterogeneity of biomedicine might be difficult to stabilise.

This paper explores one specific case of such heterogeneity - screening for developmental dysplasia of the hip (DDH) in infants in primary care. Screening for DDH brings together multiple types of uncertainty: parental uncertainties about infant development; the uncertainties General Practitioners (GPs) describe as characteristic of primary care consultations; and the inherent indeterminacies of the research evidence on diagnostic signs. A prototype checklist aimed to reduce uncertainties around diagnosis and referral in primary care. Our specific interest in this paper is in how different sources of uncertainty were surfaced and attributed through discussions about, and use of, this prototype checklist. Uncertainties arise from both ontological non-knowledge and clinicians' gaps in knowledge. However, we suggest they also arise from assumptions about other actors' gaps in knowledge. This is an example of the inevitable ‘double contingency’ (Parsons, 1991 [1951]) of social action, in which any actor is orientated not just by their own goals, but by their assumptions about the orientations, motivations and subjectivity of other actors, and by the recursive negotiations around assumptions about others' assumptions. Doing biomedicine entails managing these double contingencies as well as managing the inherent uncertainties of medical knowledge.

### 1.2 Checking infants: ‘feeling the clunk’

In the UK, the NHS's Newborn and Infant Physical Examination Programme (NIPE) (PHE 2013) 1 includes screening for problems in the heart, hips, eyes and testes at birth, then again in primary care when the infant is between six and eight weeks old (henceforth the ‘six-week check’). General guidance for GPs on how to do the six-week check advises that checks should include the baby's height, weight, head circumference, general tone and posture, lungs, and general wellbeing, and that parents should be given a range of general health promotion advice, covering topics as diverse as car safety, breastfeeding and smoking (PHE 2013).

Screening for DDH forms an important part of the NIPE. It involves a series of examinations of the infant's hips and family history to assess the risk of abnormality and decide whether to refer the infant for further investigation. Guidance for GPs is updated from time to time, following developments in the evidence base for diagnosis. The following description, for instance, was valid at the time when we conducted the research, although checking for asymmetric skin creases is no longer recommended by the NIPE guidelines:

Clinical examination involves observation of the infant for limb length discrepancy, thigh fold symmetry and any limitation of abduction. The manoeuvres of Barlow and [Ortolani] are then carried out. Barlow's test is used to dislocate an unstable but normally located femoral head. [Ortolani's] test is used to return an already dislocated femoral head to the acetabulum. Each test is considered positive if a ‘clunk’ or instability is felt as the femoral head dislocates (Barlow) or relocates ([Ortolani]) (Shorter et al., 2013)

The manoeuvres described here – and especially the resulting sensory experience of feeling ‘the clunk’ – aim to identify signs of DDH: a range of disorders in which the hip has not properly formed, or is dislocated, such that head of the femur does rest securely in the acetabulum (‘socket’) of the ball-and-socket joint.

### 1.3 Uncertain temporalities

Assessing risk of DDH early on in life is important. First, because it is common: one UK study identified 4.9/1000 newborns requiring treatment for DDH (Woodacre et al., 2016). It can affect any baby, although firstborn children, girls and those born breech are at higher risk. Second, whilst most will respond to splinting in early infancy, if diagnosed late DDH will typically require surgery, which in turn increases the likelihood for long-term problems such as chronic hip pain, difficulty walking, and early-onset arthritis (Shorter et al., 2013).

Screening for DDH – like all screening – thus raises uncertainties that relate to the inherent uncertainties of mapping future pathology onto signs in the present. Temporal uncertainty runs through the identification of infant hips that will benefit from treatment. The infant hip changes over time: the indicative ‘clunk’ may not be present at birth, but present at the six-week check (Davies et al., 2020). Not all abnormality is pathological, and there are no clear criteria for predicting which abnormality in the present will lead to problems in the future. Whole infant population ultrasound screening may reduce ‘false negatives’ (future pathology *not* identified through signs in the present) to around zero, but will identify abnormalities that might not (clinically) need correction, risking harms of over-treatment, parental anxiety and health service costs (Woodacre et al., 2014). Population ultrasound screening programmes therefore largely shift the uncertainties to other parts of a treatment pathway, given the lack of congruence between abnormality and disease (Roposch & Wright, 2007; Shorter et al., 2013). Roposch and Wright (2007: 358) summarise the multiple uncertainties inherent in clinical knowledge of DDH as relating to: “a) the identification of disease state according to different diagnostic criteria, (b) the definition of and the unclear relationship with the adverse consequences, (c) the definition of appropriate cutoff points for dividing the continuous spectrum of acetabular morphology at [ultrasound] into prognostic subgroups, and (d) the disagreement on how to define substantial risk for the predicted harm”.

There is, then, no perfect point to screen, or predictor of which infant hips will require treatment, or when: the choice of when, how and who to screen simply changes the uncertainties that have to be managed, and for whom they are most pertinent. These uncertainties reflect the inherent indeterminacies of medical knowledge (Armstrong, 2007; Fox, 1980). Yet clinicians must make decisions about how to identify infant hips that would benefit from treatment. As evidence accrues on the outcomes from different screening programmes and treatment protocols, guidelines for selecting hips for treatment evolve: yet the basic indeterminacy remains. As Armstrong (2007) notes, evidence based medicine is a strategy for reducing uncertainty that reconfigures the problem in ways that align to particular professionalising projects with medicine; but it cannot solve indeterminacy. More epidemiological evidence to address uncertainties around the optimum point to screen to reduce the risk of unnecessary treatment, for instance, cannot solve questions of value, such as how to offset risks of non-intervention and over-treatment, or at what point the benefits of whole population ultrasound screening might outweigh the costs, and to whom. For the different actors who come together in relation to screening infants at six weeks (parents, GPs, the paediatric orthopaedic surgeons to which babies are referred), different uncertainties come into play, at different temporalities, and the constellation of risks and benefits of any action cohere differently.

### 1.4 The six-week check as surveillance medicine

The six-week check is, in many ways, a paradigmatic exemplar of what Armstrong (1995) called ‘surveillance medicine’ (Armstrong, 1995), a regime of medical knowledge arising not from the observation of ill patients in hospital, but from the extension of the medical gaze across healthy populations. In Foucauldian terms, this entailed a new spatialisation of illness, which escaped the body to inhabit inter-corporeal spaces. Surveillance medicine mapped new terrains: between the normal and pathological in the creation of an always ‘at risk’ population, and across a new “temporal axis” (Armstrong, 1995, p. 492) as screening identified signs that open up uncertain possibilities of events later in the lifecourse. Armstrong notes that infants were the first targets of early twentieth century surveillance, and were its most intensely scrutinised bodies (and later minds). The six-week check enrols the entire infant population and their parents (or more specifically, usually, mothers) in the epidemiological clinic, to be assessed in terms of normality, monitored, recorded and recruited into a lifetime surveillance project. Despite its exemplar status, there has been remarkably little empirical sociological analysis of the six-week check, apart from small-scale studies of participants' views. These identified that parents found the six-week check reassuring (Roche et al., 2005) and an important ‘milestone’ of the postnatal period (Gilworth et al., 2020). Even empirical sociological work on infant surveillance more generally is limited, with a few notable exceptions, such as Grob (2008) on screening for cystic fibrosis in the USA, and Olin Lauritzen and Sachs (2001) on routine weight and height measures in Sweden.

Olin Lauritzen and Sachs' work is interesting in terms of the ways in which the technologies of surveillance medicine (in their case growth charts) might operate in terms of both generating and mitigating uncertainty. They note that the growth chart has multiple roles for parents. It suggests the constant possibility of the risk of the baby's growth veering into ‘abnormal’ trajectories; it can displace everyday parental health knowledge through focusing attention on the lines and curves of a normal distribution and the baby's place on them; but it can also offer reassurance for parents through the implied possibility of repeating measures. What is abnormal today can, potentially, be rectified, as growth is plotted into the future. Clearly technologies such as normal growth charts do not have inevitable effects: but once adopted, as May et al. (2006) suggest, they can hold together – however contingently - disparate biomedical framings, or act as ‘boundary objects’ (Star & Griesemer, 1989), operating across different logics of uncertainty.

The technology that was the focus of this study is a checklist that had been developed through a consensus process with an international group of paediatric orthopaedic surgeons to identify the most relevant criteria for diagnosing DDH (Roposch et al., 2011, 2014). The rationale for developing standardized diagnostic criteria was that there was evidence of variability in diagnosis and referral for treatment in primary care: identifying reliable and valid standardized criteria would, it was hoped, enable “general practitioners and other healthcare providers to establish the probability of DDH in a manner approaching the practice of clinical experts” (Roposch et al., 2011). For specialists, both ‘under’ and ‘over referral’ were issues, with evidence that many babies referred to hospital did not need treatment, but that some DDH was missed in the six-week check (Davies et al., 2020). In designing a consensus-based checklist, surgeons were thus addressing both the indeterminacies of scientific knowledge (through a consensus process with clinical experts) and the assumed lack of expertise in diagnosis in primary care (through reproducing this consensus in a guide for action). GPs, in turn, were invited to consider a technology that materialised specialists' assumptions about their uncertainties. Both GPs and orthopaedic surgeons were also, in different ways, orientated to parental uncertainties about infant development. We explore here the different logics of uncertainty that come together in assessing infant hips in the six-week check, focusing on how this prototype checklist surfaced not just ontological uncertainty and non-knowledge but also the double contingency of assumptions about attributions of non-knowledge.

## 2 Methods

Our data are drawn from the development phase of a study to develop and trial the checklist to aid diagnosis of and referrals for DDH in the six-week check of infants in GP practices (Roposch et al., 2020). The development phase included qualitative components to incorporate the views, experiences and practices of GPs and other healthcare staff; to explore their use of a pilot checklist; and to explore the views of parents of infants. The data generated included: 17 in-depth interviews with GPs; 14 in-depth individual or couple interviews with 16 parents; observations in ten GP practices of various sizes, during which 14 ​GPs were observed over a total of 35 baby checks; informal observation of discussions during the checklist development with healthcare and research staff from different specialist backgrounds, for example whilst the checklist was being pilot tested in a hospital setting and whilst the checklist was discussed in meetings in primary care. All were conducted in London, UK. Structured fieldnotes were written on the observations; all formal interviews were transcribed in full. Interviews and observations were all conducted by the first and second authors (SM and GG). Analysis for this paper centred on the question of uncertainty, and how it was managed by different actors.

Ethics committee approval for the study was received by King's College London University Ethics Board (MRA-17/18–6433) and Health Research Authority (18/SW/0168).

## 3 Findings

### 3.1 Parents: pervasive uncertainty, but non-knowledge about DDH

Parental uncertainty is at the heart of the story paediatric orthopaedics tells of its origins. The Ortolani test is named after Marino Ortolani, an Italian paediatrician who, it is reported, developed the test in the 1930s after a mother attending his clinic reported she could hear a ‘click’ when she washed her five-month old baby (Rang, 2000). Ortolani asked her to reproduce the noise; he went on to identify a hip dislocation on X-ray, and to develop the method of using three nappies to correct DDH. If Ortolani's test was based on a mother's concern, and her expertise in teaching him to produce, hear and feel a click, by the time Barlow published his investigations on ‘congenital problems of the hip’ less than thirty years later (Barlow, 1962), mothers had disappeared from the story. Barlow's careful studies (based, as he puts it, on ‘trial and error’) of a series of 9289 infants born in the Hope Hospital, Salford, UK examined new-borns and followed them up for a year. His findings generated knowledge on the best time to screen, and how to treat; they are the foundation for contemporary evidence-based treatment decisions. The examinations he developed (more sensitive than Ortolani's for infants in the first few weeks of life) include the one for instability of the hip now known as ‘Barlow's test’. Mothers are neither mentioned in his paper, nor are their concerns reported.

Few parents now present their babies to their GP with ‘clicky hips’. It is not parental uncertainty that brings the infant hip to the attention of the GP during the DDH check, but the universal surveillance of the NIPE. Indeed, in our study, few parents reported any awareness of DDH as a developmental condition in general, or as something of concern in relation to their infant. In the here-and-now, infant hips rarely presented to parents as problems, and DDH was, largely, an area of parental non-knowledge. Parents only reported the possibility of DDH as a known unknown if it later became salient, for instance where the GP had referred the infant on for further investigation.

Mother: *well I was not worried at all, until I got to the* [hip ultrasound] *clinic.*

Father: *I think we weren't worried about it, I think you're right, we weren't worried about it at all up until that point.*

Mother: *I'd never heard of this condition, I'd never seen it.* (Parents-3)

If parents, in general, had little knowledge of current or potential future problems of infant hips, it was apparent that GPs did not expect any knowledge. In the six-week checks observed, GPs typically asked for parental input into a number of issues on infant development (such as whether the infant yet smiles); but we observed no questions directed to the parents around their experiences of their infants’ hips, nor any mentions by parents of signs of DDH.

For the parents and carers bringing the infant to the clinic, the six-week check was often described as a ‘milestone’ encounter, during which they received, and expected to receive, confirmation and certainty that both they and the baby are ‘doing ok’ (Gilworth et al., 2020). Rather than eliciting specific uncertainties around their infant's health, anticipations of the six-week check were of a ritual to mark holistic reassurance that the baby was normal and healthy:

*I was glad that it was happening really because I quite like that, I quite like that reassurance really. So I sort of felt quite positive about it.* (Mother-10)

*I just know they do a thorough check of everything … after each bit I just say thank God that he's healthy and that he's actually OK, and he passes. (Mother-6)*

That the screening examinations were often described as something the baby ‘passes’ suggests the symbolic value of this ritual for parents and carers, and the underlying uncertainties that suffuse the early weeks of infancy. However, interviewed shortly after their infants' check, parents rarely reported having had any detailed expectations of clinical examinations that would be undertaken, or of the specific disorders that would be screened for. Few articulated in any concrete terms what they expected from the six-week check; many simply noted this as an area of non-knowledge:

*I haven't given it much thought about exactly what's going to go on.* (Mother-4)

The six-week check, even for experienced parents, was largely a ‘black box’. Parents did not consider the details of clinical examinations undertaken as something that they ought to have known about, neither did they recognise this lack of knowledge as problematic. Their assumption was that a knowledgeable and skilled GP would undertake a set of routine tests to assess and reassure them about their infant's general health and normality. The tests for DDH (like other specific tests done) were neither specifically anticipated, nor articulated as an issue of concern.

This is not to say parents did not report uncertainty about their infants' health. Indeed, many reported considerable uncertainty about their infant's wellbeing and their own coping as parents. However, these uncertainties centred on other aspects of care for their new babies. Some GP practices, for instance, carry out the first vaccinations of the infant at the same time as the baby checks. In contrast to the lack of articulated uncertainty about screening examinations, vaccinations were typically associated with detailed and specific uncertainties:

*I'm much more focused on the vaccinations than I am on the check … like how many Calpol doses, is she going to get a fever, is it going to spike, should my husband take time off, is the night going to be really bad afterwards?* (Mother-4)

Only when prompted specifically about the hip examination did this participant reflect that she should perhaps have considered it, given that her husband's sister had experienced DDH. However, in the interview, this recollection of ‘knowing’ at one level (that is, having ‘heard of’) about DDH did not translate into a refocus on DDH as a concern for her, even in retrospect.

More generally, parents described early parenthood as a time of high levels of trust in the healthcare system, in biomedical knowledge, and the status of their GP as an expert in that knowledge, even when this trust in healthcare expertise was not habitual practice in the rest of their lives. Accounts of early parenthood were of a time suffused with questions and uncertainties; healthcare professionals were referred to as key actors for reducing that uncertainty. These parents, although not patients, were perhaps positioning themselves as such in classic Parsonian terms: in need of competent help and reassurance, and willing to put faith in biomedicine. This taken-for-granted trust in health care professionals was most visible in its absence, when disrupted, as illustrated in this short case from the study.

Renata (Mother-2) was one of the few in our sample whose baby was referred for suspected DDH. She described feeling ‘failed’ by the NHS at multiple points during the diagnosis and treatment of her baby. Retrospectively, she described herself as having a high level of agency and responsibility in her self-care and interaction with the health system before her baby arrived, yet reported that this was suspended in the immediate period of time following the birth of her baby due to the emotional labour and exhaustion involved with learning to parent a new-born: *‘normally I would be on top of things but I was just so thrown by having a baby’*. In these excerpts from her interview, she describes how this intense focus on the birth precluded consideration of potential post-natal issues:

*Up until this point to be honest, even preparing for the birth I hadn't really looked at after the birth and what needed to be done to kind of keep children healthy or check children after they were born. I'd been worried about how the birth would go … I actually didn't know what checks were supposed to happen … I hadn't looked it up in advance*.

*I was just going along with what I was told to do*

*I was in this weird mode of just taking it on trust, or just trusting the healthcare system so much that I wasn't doing my own research, and it's so bizarre because I'm someone who works in healthcare … I don't know what happened to me.*

Renata's baby was referred for a hip ultrasound from the hospital soon after birth, but she reports that this did not prompt concern for her; as for other parents, the immediate concerns of managing with a new baby were simply more pressing:

*it didn't hit me as, oh no there's something wrong, like not at all … it is really full on, just feeding them and being up all night and just being exhausted all the time.*

Uncertainties around hips, then, are neither foregrounded by parents (even those who might be particularly attuned to DDH as a potential issue) nor considered (by themselves or GPs) as something that ‘should’ be high on their agenda in the infant's first weeks, given the multiple other pressing needs of the time. Even parents who presented themselves as knowledgeable and generally agentic about health care described early infancy as a point where one has to largely trust health providers (even if in retrospect this appeared, as for Renata, ‘weird’), rather than be orientated to identifying more potential areas of uncertainty.

In considering this parental trust and non-knowledge, some hospital clinicians suggested that heightening uncertainty for parents might be beneficial. Evoking the centrality of the mother's experiential knowledge in Ortolani's history, this group of hospital clinicians, for instance, saw greater parental knowledge about DDH as a potential backstop against what was positioned as the ‘ignorance’ of primary care:

*[the clinicians] described having quite a few experiences with late referrals, where the parents had been concerned about their babies' hips but had been reassured by the GP, wrongly, that there was nothing wrong with them. [They] thought cases like this might have been solved if parents ‘had more education’ that they could use – because despite the lack of awareness around DDH, it is the most common orthopaedic condition in babies.* (Hospital Clinic Fieldnotes, 241018)

However, although Renata, in retrospect, expressed regret at taking things ‘on trust’, trust is perhaps a requirement of effective health care, at least in this Parsonian model (Parsons, 1991 [1951]). Greater knowledge, rather than reducing uncertainty, simply shifts the uncertainty from what best to do, to whether the health care provider is competent or not. Formal knowledge can erode taken-for-granted trust (Legido-Quigley et al., 2014), and thus potentially increase anxiety. GPs largely saw more knowledgeable parents in this sense: as likely to generate a dysfunctional, rather than functional, uncertainty. One GP was explicit on these risks:

*If you did, as a parent, know how many things there could be wrong, would you be like freaking out? Because there's so many things that you're looking for that could be wrong but it's such a quick exam, would you start, would it cause more anxiety? I don't know.* (GP-3)

For parents, then, uncertainties relating to early infant health rarely focused on the (potential) problem of DDH. Other concerns were more pressing, and they largely expected the six-week check to be a ritual of reassurance around infant ‘normality’ rather than screening for abnormality. Trust in biomedicine was high, and if parental uncertainty about a ‘click’ is at the heart of paediatric orthopaedic history, it has largely disappeared from its present.

### 3.2 The primary care context: a consultation designed to elicit uncertainties

If parents saw the six-week check as offering holistic reduction of uncertainty around infant health and normality, this was also largely how GPs also described it, if in more clinical terms. GPs described the broad aim of the baby check as ascertaining whether the infant is ‘*anatomically normal*’ (GP-2), which involves examining multiple aspects of the infant's body from ‘*top to toe*’. However, this infant check is often combined with the routine postnatal check of the infant's mother, which includes birth complications, stitches or scars, and discussions about levels of social support and observations of the interaction between mother and infant to deduce risks of postnatal depression. The check for DDH is just one element of a complex and often open-ended consultation. Baby checks are unusual within UK general practice settings, in that in many practices they are perceived as *‘unrushed’* appointments, and ‘*sought after’* (GP-3) work because of their ‘*more relaxed’* nature. In the observed practices, the time allocated ranged from 15 ​min to up to an hour per consultation, compared with the usual allocation in most UK general practices of under 10 ​min per consultation. This is, then, a rare clinical encounter, operating not just as a routine screening, but also as a consultation where a range of concerns could be presented. There is time for the clinician and parent(s) to settle, undress and dress the baby, perform the required examinations, record the findings on both computerised records and paper patient-held records, and provide space for the parents to bring up problems, introduce other topics, and open up discussion in ways that are impossible in the more typical UK primary care encounter.

One other contrast to a typical contemporary GP consultation in UK primary care, which proceeds largely by verbal question and answer, was the overtly physical and sensory nature of the baby check. To examine, discuss and observe the baby's body and demeanour in the six-week check, GPs use all their senses. Sensory evidence emerged from overlapping attunements to the infant and mother. As this GP explains, examinations entail a simultaneous listening to and watching the mother, and listening to, watching and feeling the baby.

*I've been able to sort of eyeball the baby a bit … and I've also heard by then whether the birth was straightforward … I always listen to their heart first because that's the one thing I want them to be really, really quiet for. I've already been able to observe that they're not struggling for breath or a funny colour, just looking while we're chatting … I hoick her out, sort of talking a while just to sort of get a feel, …it's important to handle them just to get a feel for tone … then I make eye contact and check that they will fix and follow … and all the time you can sort of see if they're moving normally and symmetrically.* (GP-4)

This examination is an embodied interaction between physician and baby, with material technologies employed largely to enhance the senses, such as a light (to check for the red reflex in the eyes), a stethoscope (to aid listening to the chest and heart) and scales to weigh the baby. In addition, a number of technologies to aid diagnosis and referral already participate in the encounter. These included existing computerised checklists (reportedly different across practices, some with pop ups on computer screens to prompt for particular signs), and (in all practices) the ‘Red Book’, a material, A5 size book held by the parents. This Red Book (also called the Personal Child Health Record) is issued by the National Health Service shortly after the birth, and contains records of the infant's physical measurements, growth and examinations, vaccinations, some guidance for parents, and contact information for various health professionals. Parents are expected to carry their Red Book to all the infant's appointments with the various health professionals that they consult with in the first year or so of life, and the GP is expected to record measurements in the Red Book as well as on their practice computer systems. The Red Book was an object shared by parents and clinicians, and which reportedly prompted action – particularly acting as an aide memoire for some GPs in conducting the examinations:

*I just go through the Red Book and they've got, I don't know how many things there are, but hips and I can't remember if it comes in with feet as well or if it's one on its own, but there's a standard list of things and you for each one to say whether it's normal or abnormal or you need further assessment or that kind of thing. So [I do the examinations] then … go back to the Red Book and just double check I've gone through everything.* (GP-2)

The diagnosis of DDH relied primarily on the embodied, sensory expertise of the GP, rather than material technologies. The GP must move the baby's limbs, using just the right amount of pressure, and feel and listen for an abnormality: a distinctive ‘clunk’.

### 3.3 Gaps in experiential and embodied knowledge: recognising clunks

A key source of acknowledged uncertainty for many GPs was their own lack of experience in feeling this ‘clunk’. In medical school, they recalled learning to do the hip examinations using a rag doll, which did not have the feel of a real joint. The feeling and sound of the ‘clunk’ of a dis- or relocated hip might well be something they had never felt nor heard before. For less experienced GPs in particular, this acknowledged gap in clinical expertise was on occasion explicitly attributed as a cause of ‘being on the safe side’ in their referral practices: uncertainty was shifted to specialists.

*I've never felt one where I've been like that, that's definitely it, so I would always err on the side of caution just because I'm not that experienced, and anything that felt a bit clicky I would always refer for a scan.* (GP-2)

*You know, you hear about the clunk and the clicks and things, but the thing is you've never come across it* (GP-16)

Teaching others to do the tests relied on transferring embodied knowledge, such as the most effective way to hold the baby, or how to calibrate the precise amount of pressure to use when manipulating infant limbs by ‘*doing it firmly enough but not too hard’* (GP-4). This tacit knowledge, rooted in experiential expertise of feel and touch was difficult to articulate verbally, and thus skill in ‘feeling’ a clunky hip was difficult to acquire without hands-on experience. However, it was a feeling widely cited as ‘never forgotten’ once experienced. A single clinical encounter was enough to learn the distinctive feel of a positive test:

*It's so hard to describe but it's a feeling thing … Until you've felt it I think there's a real worry that you* [might miss it]*, once you've felt one, you'll say oh you'll never forget it now … it's a sort of very odd feeling.* (GP-4)

*[feeling it] was quite obvious so yeah, I'm quite glad to have felt one. It was good experience and it also makes you think that you probably haven't missed it before because I did that exam the same way I would do it.* (GP-6)

For GPs, learning to ‘feel’ the clunk is typical of the wide range of competencies they must acquire once they begin their GP training. Learning is by apprenticeship as much as through formal courses, and opportunities to acquire specific skills or experiences relating to infant screening depended on the specificities of the practice. The baby check in general was something that one just ‘did’, and then acquired expertise along the way. On conducting their first hip exam, GPs were lucky if they had even been shown one first:

*I was speaking to another one of my doctor friends today about the fact I was going to do this interview and she was like, “I don't really do six-week checks, do you need to do a course to be able to be qualified to do them?” It's like, no, you just learn.* (GP-3)

*We're essentially going on having been taught once. It's the see one, do one, teach one approach in medicine*. (GP-7)

### 3.4 The checklist surfaces limitations in one's own medical knowledge

Conducting six-week checks swiftly became routinized, even for those who may have never felt a ‘clunk’. However, discussing the prototype checklist for referring suspected DDH surfaced new uncertainties for GPs. The checklist was an attempt to reduce uncertainty arising from gaps in expertise, through consolidating expert consensus in a way that would enable a ‘non-expert’ (the GP) to reliably replicate the decision making of experts. It consisted of eight items, with a box to check if the GP found them present: family history of DDH; a positive Barlow test; a positive Ortolani test; asymmetry in hip abduction of 20° or more; abduction of one or both hips limited to 45°; any leg length discrepancy; presence of torticollis; or presence of clubfoot or other fixed foot deformities. The initial format was a paper document, which was shared during interviews and observations of the six-week check, or presented to GPs for discussion on the researcher's laptop, and then observed ‘in practice’ as a paper form to be completed.

A first area of uncertainty surfaced was the gulf between GPs' own framing, and the uncertainty they imagined as located within the orthopaedic specialists who had designed this checklist. A physical examination that generally took the GPs about 30 ​seconds, as one embodied, fluid and sequential movement, was separated out and formalised in the material checklist, with components described in technical detail which may never have been overtly known (‘rotating the hip to 45°’), although tacitly understood. Reading a technical description on a piece of paper or as a document on a computer reframed this known, but tacit, knowledge as a newly-acknowledged domain of non-knowledge:

*I showed them the [draft] checklist on my laptop and they pored over it … They … discussed each point on the list, laughing at how different it felt seeing it all laid out like that compared to the feeling of doing it in the baby clinics. They all laughed at the point which describes manipulating the hip to 45degreesand said this was ‘obviously written by an orthopaedic surgeon’. They also discussed the Barlow and Ortolani tests, like the other GPs I have spoken to, and one of the partners said she refers to these movements of the hips as ‘Barlani’.* (Primary Care Practice Meeting Fieldnotes, 081118)

Second, a checklist focusing on one single part of the infant anatomy materialises, for GPs, an important area of specialists’ assumed ignorance about the orientations of primary care. The checklist not only both broke down the hip examination into discrete points, but it also focused solely on one element of the whole consultation, the hip examination. This jarred with how GPs thought of and performed the consultation:

*I guess it's taking into context that examining the hips, although it's an important part of baby checks, so much of it is an important part of the baby check and the mum check, and no, we don't want to miss a hip, but, equally we don't want to miss, you know, an eye or miss a femoral pulse.* (GP-7)

This GP went on to suggest that technologies such as this checklist were often seen by primary care as coming from ‘*single issue pressure groups’,* which would advocate for their specialist area, and inevitably want GPs to focus on that. Here, a technology arising from specialists, focusing on one test or disease, surfaced GPs' attributions of specialist ignorance; they could not be knowledgeable about the realities of primary care if designing a checklist for one discrete condition, as this was *‘the total opposite of what general practice is … it's the whole of the community’.*

Third, the individual items on the list surfaced new uncertainties in technical knowledge: or at least ones that had not figured in their observed practice, or the interview, until this point. As they went through the items, some GPs began reflecting on and dissecting their examination techniques, questioning whether they were doing it correctly. A common example was the names of two key tests: the Barlow test (to see if the head of the femur could be dislocated) and Ortolani test (to see if the head of the femur was dislocated). The initial prototype checklist had no prompts as to which test was which, and many GPs reflected a lack of confidence that they knew this:

*I can't remember which way round they are.* (GP-3)

*I probably mixed them up before I saw the video [to train GPs in using the checklist]! [laughing] And do you know, it's like when you're teaching medical students, erm, they're more likely to say to me ‘oh, that's the Ortolani, that's the Barlow’ because for them, they have to know this stuff. In reality it doesn't really matter which one is the Barlow and which one is the, you know, it doesn't matter. (GP-15)*

Although they were perfectly capable of doing these tests in practice, as the GP above, who calls it the ‘Barlani’, suggests, having to tick separate boxes on a checklist made non-knowledge newly pertinent. The key issue for making a referral is that one of the tests is positive: knowing the respective names of the two tests is not knowledge that makes a practical difference to clinical competence. It is, however, knowledge that makes a practical difference to being able to complete a checklist.

Finally, the checklist surfaced issues around the indeterminacy of the evidence base – at least over time. Checking for asymmetric hip creases was not an item on the checklist, but was something some GPs did do routinely, and (at the time of the study) still listed in much guidance for doing the check. Discussing the checklist elicited uncertainties around the diagnostic relevance of this, and over whether that uncertainty was located in the evidence base or within the GP's own expertise:

*GP: I think there might have been a hip crease asymmetry at some point [as a reason for referral] ….*

*I: It's interesting because that, other people have said about the hip creases but that's not actually on the checklist?*

*GP: It's on the, I think one of them was asymmetry, isn't it? Oh, I can't remember what it says …. Maybe they've validated that one, and found it not useful?* (GP-2)

### 3.5 Evidence is not enough to reduce referral decision uncertainty

Surfacing new uncertainties about their own non-knowledge, and the double contingency of their assumptions about specialists’ assumptions about that non-knowledge, did not mean that GPs rejected the idea of a checklist deriving from orthopaedic colleagues. Indeed, the logic of reducing uncertainty through clarifying thresholds for referral was appealing for some, who explicitly flagged the utility of a technology to aid decisions that were otherwise difficult to both make and potentially justify:

*It might be quite useful to have like a go-to.* (GP-3)

*Sometimes you can get a bit, you know, ‘I've done this so many times, you know, it's fine, I'm not going to miss anything’ and you know, a jolt like that to remind you it can be missed, I think is very useful …..[talking about checklist] So I usually, I had a look at it before they [parents] come in, just to remind myself, just to remember to go through all those points … then as I'm coming to the end, I will make sure I've ticked everything that needs to be ticked.* (GP-15)

However, uncertainties around referral decisions were not, in practice, so easily reduced to a tick-box. In observations of the initial trial of the checklist (as a paper document), few GPs did refer to it during the consultation; most completed it after the parents had left the room, or as the baby was being dressed. Despite its potential to act within the consultation, as suggested by some GPs, its embryonic material form as a paper document did not appear to enable this. Moving between the examination couch, the desk with a computer and the sink, few GPs reached for the new form. Indeed, on at least one occasion, the paper checklists were collected from reception and completed at the end of the clinic, rather than referred to during the consultation. The new technology, focused on one small part of screening, could not be straightforwardly adopted within a routinized, largely fluid existing consultation.

Beyond the challenges of normalising a new technology were some more fundamental ways in which the checklist failed to reduce uncertainty. These related to the relationship of primary care (with its undifferentiated workload), the ‘population surveillance’ of the six-week check, and the assumed roles of other clinical specialties. The checklist was a technology aimed at encapsulating specialist knowledge, and transporting it to primary care, where (it was assumed) non-specialists who may be relatively inexperienced could then benefit from more ‘certain’ evidence at their disposal as a tool within screening. Thus, one specialist from the hospital team reflected on their experience with local GPs:

*[the clinician] mentioned that from their perspective as [associated healthcare professionals] that they didn't think GPs were confident with their examinations, and that maybe the GPs just don't see enough babies to be that interested - said, ‘it's too sporadic for them’.* (Hospital Clinic Fieldnotes, 241018)

The rationale behind the development of the checklist was to help GPs make more appropriate referrals through providing a series of prompts for the necessary checks, and when to refer, thus replacing ‘uncertainty’ on the part of GPs (which was passed on to the hospital team) with a more ‘certain’ referral threshold (leaving specialists to deal with ‘more certain’ problems). However, this attempt at uncertainty reduction in primary care was difficult to reconcile with GPs' attributions of specialist referral as the legitimate backstop provider of uncertainty reduction. First, retrospectively, there was an assumption by some GPs that the first anatomical check performed within the first 72 ​hours (PHE 2013) of life at the hospital after the infant is born would be the proper place where competent hospital paediatricians would identify any major problems:

*I definitely am aware that babies born in this country have had a check before they've left hospital, a new baby check, so you kind of hope that a lot of things have been picked up at that stage.* (GP-13)

Second, for others, who did recognise the need for a thorough six-week check to identify later-emerging problems, referral to specialists was the legitimate route through which acknowledged uncertainties at the primary care level *should* be reduced. The (assumed) logic of moving ‘specialist’ decision making to the primary care setting was recognised by some GPs, but it was also resisted, as making inappropriate assumptions about their own referral logics. Whereas the logic of the checklist was (it was assumed) based on ‘accuracy’; their referral logic was based on ‘safety first’. Here, for instance, GPs' comments gesture to their assumptions about the specialists' assumptions: that GPs are ‘over-referring’ due to uncertainty:

*Too many and I'm probably over-referring and too few and I'm under-referring, what's just right? … if that's what [the specialists] mean, that people waste [their] time in clinics by sending [them] hips that are okay, well that's sort of what [they are] there for.* (GP-4)

*our biggest worry would be to miss something … I mean, certainly I would consider that my role [to refer]. I think for a six or eight week year old we're not referring that many of, why take the risk? …. it only takes one patient that you miss for it to be a terrible outcome, for instance. So that's why we're always on that side rather than the other side. (GP-14)*

In defence against the *assumed* logics of a distant, unknown paediatric orthopaedic team, materialised in the prototype checklist, GPs stressed their care in making appropriate referrals. In general, they argued, referrals are not made lightly, but result from balancing different factors, including patient demand and health service resources as well as evidence-based clinical need, sometimes in consultation with others from the primary care team: ‘*every referral is essentially discussed with another GP or another doctor … we try to avoid unnecessary referrals’* (GP-7).

## 4 Discussion

We have described multiple sources of uncertainty that surround screening for DDH in primary care. Ontological uncertainty arises from inevitable limitations in the research evidence: DDH remains elusive, with debate about its aetiology, incidence, the best point to screen and appropriate treatment thresholds (Davies et al., 2020; Roposch & Wright, 2007; Shorter et al., 2013). These uncertainties matter for all actors. An attempt to consolidate consensus guidance around a checklist for the signs of DDH at six weeks held the promise of reducing uncertainty for one set of actors (GPs), but it also surfaced new ones. These arose as GPs recognised limitations in their own sensory expertise (in ‘feeling the clunk’) and formal knowledge (of the names of tests, or of the latest evidence based guidance), but also in their questioning of the assumptions of other actors (specialist paediatric orthopaedic surgeons) about their practices. Discussing the checklist foregrounded the different logics underpinning these sources of uncertainty, with a routinized checklist sitting uneasily with the requirements of primary care. The checklist entered an already hybrid encounter, which combined a ritual of reassurance for parents in the here-and-now, with future-orientated infant surveillance, and screening for DDH in the context of a holistic primary care consultation.

The proposed checklist was a welcome intervention in some senses, which would (some anticipated) help with an inherently uncertain decision – whether to refer. In terms of a stochastic framing of uncertainty around the relative risks of under and over referral, clear criteria and a decision tree for when to refer would clarify the threshold. In other senses (and for other GPs), however, the checklist failed to articulate with their own primary sources of uncertainty, which could only be reduced through further clinical experience, specifically sensory and embodied experience, summarised in this case as learning to ‘feel the clunk’. The proposed technology was potentially uncertainty-reducing at the level of intellectual clinical knowledge, but not in terms of embodied clinical expertise, which was the more pressing issue for those who had no experience of ‘feeling the clunk’. The logic of a ‘more reliable’ referral decision also articulated poorly with the logic of a ‘safety first’ referral decision.

Timmermans and Angell (2001) documented the different orientations to evidence based medicine among paediatric residents from two medical programs in the US; these differences were reflected in the GPs in this sample, who differed in their general orientations to the usefulness of guidelines and checklists. To an extent, these mapped to divisions between what Jones and Green (2006) called the ‘new’ general practice, with its emphasis on continuing medical education, and utilising the best available evidence, and more traditional models, which tend to focus on patient-centred medicine, enduring patient doctor relationships over time, and the value of embodied experience, rather than codified medical knowledge. More experienced GPs had, in general, accommodated to what Fox (1989:84) described as the “enormity” of medicine and “the impossibility of commanding all of it”. A new checklist could be welcomed as an ‘update’ on clinical knowledge: but there was no sense in which, it itself, it could reduce the inevitable uncertainties of dealing with the unpredictable, holistic and contingent problems that might arise from a six-week check of an infant.

Focusing on the point at which the checklist was still in prototype form enabled the contestations around these sources of uncertainty to be more visible than they might otherwise have been. Specifically, debates around ‘whose’ uncertainty was the target of reduction surfaced the double contingency of communication. Parsons described the element of ‘double contingency’ that is always present in social systems, whereby “contingent alternatives not only of ego's actions, but of alter's, and the possible permutations and combinations of the relations between them” (1991[1951] p6) orient social action. In our case, actors were orientated not just to their own gaps in knowledge (or those in biomedicine) but to their expectations of how other actors constituted their ignorance. Thus, the paediatric orthopaedic surgeons were oriented to the (assumed) technical uncertainties of GPs, who lacked skills in conducting correct examinations, knowing the signs of DDH, and making appropriate referral decisions. GPs were recursively orientated to this assumption, in defending their more holistic examinations and referral practices as rooted in a rather different logic of uncertainty. They were also orientated to an assumption that specialists were ignorant of their holistic approach to the six-week check. Largely missing from the orientations of both were the uncertainties of parents about infant hips. These existed only as assumptions about parental ignorance: that it was necessary as an uncertainty-reducing state (in the accounts of GPs); or it was a problem (for specialists), in that it reduced the likelihood of parents' anxieties acting as a backstop for timely action on diagnoses. Parental uncertainties about ‘clicky hips’, or other abnormalities, appear to have been successfully delegated to the technologies of infant surveillance.

The double contingency of acting in relation to assumptions about the orientations of other actors was particularly explicit in our example, as our data come from a context in which we were asking GPs about a checklist they assumed (correctly) as generated by another clinical speciality, paediatric orthopaedic surgery. However, the double contingency of uncertainty attribution is likely to be a broader issue in medicine. Routine clinical practices such as prescribing, making referrals, or managing chronic illness, might be orientated not by a clinician's own recognition of the limits to medical knowledge – or their own limited knowledge – but by their assumptions of the uncertainties of others, such as patients' needs for reassurance. In exploring patient expectations of antibiotic prescriptions, for instance, Boiko et al. (2020) point to the reflexive ‘expectations of expectations’ between patients and prescribers, with each actor orientated to what they assumed to be the attributions of uncertainty of the other.

If, as in Parsons' account, double contingency is classically a problem of social actors, who must reflexively consider the potential motivations of others, then our case also suggests that non-human actors, such as checklists, can play a role. The prototype checklist for DDH materialised not a stable boundary object, but rather the different logics of uncertainty across clinical specialities and parents. As May et al. (2006) suggest, all technogovernance has inherent contradictions, and in a primary care arena these include the lack of coherence between the logics of ‘patient centred’ medicine and evidence-based guidelines of the kind materialised in our checklist. Our case involved parents, not patients, and a consultation focused on screening rather than chronic illness management, but a similarly hybrid biomedicine was apparent, in the different biomedical knowledges brought to the infant hip examination in the six-week check. These were stabilised to the extent that infant hips were checked, referrals were made, and parents were reassured. Yet the checklist not only also generated new areas of uncertainty and non-knowledge, but also highlighted the relationality of these uncertainties. Uncertainty and non-knowledge are distributed across different stakeholders in the process of screening for DDH, and each actor is orientated to not just their own uncertainty, but to their ‘assumptions about the assumptions’ of others. Just as the technologies (such as here, the ‘Red Book’) had an agency in the encounter (prompting actions), the new checklist was also anticipated (by the orthopaedic surgeons and some GPs) to exercise agency, and change practices. However, unlike the Red Book, the checklist could not yet stabilise the inevitable heterogeneity of biomedicine. Instead, it revealed existing uncertainties, created new ones, and – importantly - orientated key actors to the uncertainties of others.

## Ethical statement

All participants in interviews and observations were provided with an information sheet, time to consider and answer questions, and provided written consent to participate.

Ethics committee approval for the study was received by KCL (MRA-17/18-6433) and NHS (18/SW/0168).

## Funding

This project is funded by the 10.13039/501100000272National Institute for Health Research (10.13039/501100000272NIHR) Programme Grants for Applied Research (project reference RP-PG-0616-20006). The views expressed are those of the author(s) and not necessarily those of the NIHR or the Department of Health and Social Care. The funder played no part in the study design; collection, analysis and interpretation of data; the writing of the report; or in the decision to submit the article for publication. Judith Green is supported by a 10.13039/100010269Wellcome Trust Centre Grant 203109/Z/16/Z.

## Author contributions

Sarah Milton: Conceptualization; Data curation; Formal analysis; Supervision; Investigation; Methodology; Project administration; Writing - original draft; review & editing.

Gill Gilworth: Data curation; Formal analysis; Investigation; Methodology; Project administration; Writing - review & editing.

Andreas Roposch: Funding acquisition; Methodology; Supervision; Writing - review & editing.

Judith Green: Conceptualization; Formal analysis; Supervision; Funding acquisition; Methodology; Writing - original draft; review & editing.

## Declaration of competing interest

The authors declare that they have no known competing financial interests or personal relationships that could have appeared to influence the work reported in this paper.

## Footnotes

1 Guidance was updated in 2021, after the fieldwork reported here, with changes to the physical examinations recommended: https://www.gov.uk/government/publications/newborn-and-infant-physical-examination-programme-handbook/newborn-and-infant-physical-examination-screening-programme-handbook#screening-examination-of-the-hips.
